# Extraction and Characterization of Extracellular Proteins and Their Post-Translational Modifications from *Arabidopsis thaliana* Suspension Cell Cultures and Seedlings: A Critical Review

**DOI:** 10.3390/proteomes4030025

**Published:** 2016-09-01

**Authors:** Mina Ghahremani, Kyla A. Stigter, William Plaxton

**Affiliations:** 1 Department of Biology, Queen’s University, Kingston, ON K7L 3N6, Canada; m.ghahremani@queensu.ca (M.G.); 0ks11@queensu.ca (K.A.S.); 2 Department of Biomedical and Molecular Sciences, Queen’s University, Kingston, ON K7L 3N6, Canada

**Keywords:** cell wall proteomics, secretome proteomics, post-translational modification, glycosylation, phosphorylation

## Abstract

Proteins secreted by plant cells into the extracellular space, consisting of the cell wall, apoplastic fluid, and rhizosphere, play crucial roles during development, nutrient acquisition, and stress acclimation. However, isolating the full range of secreted proteins has proven difficult, and new strategies are constantly evolving to increase the number of proteins that can be detected and identified. In addition, the dynamic nature of the extracellular proteome presents the further challenge of identifying and characterizing the post-translational modifications (PTMs) of secreted proteins, particularly glycosylation and phosphorylation. Such PTMs are common and important regulatory modifications of proteins, playing a key role in many biological processes. This review explores the most recent methods in isolating and characterizing the plant extracellular proteome with a focus on the model plant *Arabidopsis thaliana*, highlighting the current challenges yet to be overcome. Moreover, the crucial role of protein PTMs in cell wall signalling, development, and plant responses to biotic and abiotic stress is discussed.

## 1. Introduction

The plant cell wall is a dynamic matrix of polysaccharide networks that provides structural support and serves as a first line of cellular defense against abiotic and biotic stressors. The primary cell wall is mainly composed of hemicelluloses intertwined with cellulose microfibrils and pectins, whereas the secondary cell wall can contain wax, lignins, and cutin [1,2,3,4,5]. While polysaccharides can represent greater than 90% of the primary cell wall mass, there is a burgeoning interest in identifying and characterizing the cell wall proteins (CWPs) that account for the remaining 5%–10% [3,5,6,7,8,9]. CWPs govern the changes in polysaccharide composition and orientation during plant development and acclimation to environmental cues such as light, gravity, and hormone signalling [1,2,4,10]. During cell elongation, the action of CWPs such as hydrolases, transglycosylases, lyases, peroxidases, and expansins is required to loosen the polysaccharide matrix [4,10,11,12,13]. Likewise, following cell elongation or during conditions of stress, CWPs can cross-link polysaccharides, thus rigidifying the cell wall [4,7,10]. CWPs also function in a signalling capacity, relaying information from the exterior environment and mitigating signal transduction cascades to achieve the appropriate intracellular response to external stimuli [5,14,15,16,17,18,19,20]. Understanding how and when CWPs are secreted and activated could provide great insight into the dynamic nature of the cell wall and how plants are able to respond to external stressors and stimuli. Of particular interest are the post-translational modifications (PTMs) of CWPs, as they can play a crucial role in determining a protein’s activity, localization, and/or interaction with other proteins [1,21,22]. In vitro, proteins may appear to have redundant functions, but in vivo PTMs can confer non-redundant function due to variance in activity and cellular targeting [21]. The majority of CWPs undergo some sort of PTM, the most common modifications being proteolytic cleavage, glycosylation, phosphorylation, glypiation, and the hydroxylation of proline to form hydroxyproline [22,23,24]. Not only can these PTMs target proteins to the cell wall, but they may also hold integral roles in cellular responses to biotic and abiotic stress, thus contributing to signalling processes [6,24,25,26]. Identifying CWPs and their associated PTMs under varying developmental and environmental conditions will ascertain potential targets for more directed, downstream proteomic analyses, allowing for a more detailed picture of CWP function and regulation to emerge.

The model plant *Arabidopsis thaliana* leads the field of plant cell wall proteomics [1]. However, less than one third of the approximately 5000 *Arabidopsis* genes encoding proteins predicted to be targeted to the secretory pathway have been identified [3], as there are several challenges related to CWP extraction procedures:

(1) The cell wall, having no discrete membrane, is not a bounded organelle [1,4]. Instead, the cell wall may be viewed as a continuum, which makes it difficult to isolate only the cell wall and apoplastic fluid proteome without contamination by intracellular proteins. During extraction, many intracellular proteins can bind to the charged polysaccharides of the cell wall matrix and be mistakenly included in the cell wall fraction [4,27]. In addition, some bona fide apoplastic proteins may not associate directly with the cell wall, and therefore be discarded as part of the intracellular fraction [4].
(2) As CWPs only comprise 5%–10% of the cell wall mass, challenges arise in enriching the CWPs, particularly since they can be embedded within the polysaccharide matrix. Some CWPs interact with the cell wall via non-covalent linkages, such as ionic, hydrophobic, or van der Waals interactions, while other CWPs can be covalently cross-linked with the polysaccharides, thus forming insoluble networks [1,4,5,28,29,30]. To date, no single protocol has successfully extracted all CWPs due to the high degree of heterogeneity by which they can associate with other cell wall components [27]. In addition, the plant cell wall varies both spatially and temporally, further confounding the elucidation of a complete cell wall proteome [1,4].
(3) Once CWPs have been successfully extracted, it can be difficult to separate and identify them. Most CWPs are basic, making the conventional two-dimensional polyacrylamide gel electrophoresis (2D-PAGE) method of protein separation less effective [1]. Furthermore, low-abundance proteins or small peptide hormones are generally not detected using this traditional method of protein separation; as such, many important CWPs may be overlooked or missed completely. Additionally, PTMs of CWPs can complicate both protein separation, and protein identification and characterization using bioinformatic tools [1,3,5,6,22,23,24,26].
(4) Finally, studies aiming to identify the extracellular proteome are often plagued by so-called non-canonical CWPs [1,31,32]. Despite evidence of minimal intracellular contamination, these proteins lacking the classical signal peptide that would target them to the secretory pathway persistently appear in CWP extracts, with an estimated 40%–70% of proteins identified in secretome studies falling within this non-canonical grouping [32]. Some of these proteins may indeed be intracellular contaminants; however, recent evidence suggest that a plant-specific exocyst-positive organelle may be responsible for the unconventional secretion of certain CWPs [5,32,33]. Leaderless secretion of some proteins also occurs in both mammals and yeast, and may be common to all eukaryotes [31].

While the cell wall proteome has been studied in a wide variety of plant species including *Oryza sativa* [34,35], *Zea mays* [36,37,38,39], *Triticum aestivum* [40], *Solanum lycopersicum* [41,42,43], *Brassica napus* [44,45], *Medicago sativa* [46], *Glycine max* [47], *Medicago truncatula* [48], *Nicotiana tobacum* [49], *Populus deltoides* [50], *Vitis vinifera* [51,52], *Cicer arietinum* [53,54,55], *Brassica oleracea* [56], *Linum usitatissmum* [57], *Solanum tuberosum* [58], *Hemileia vastarix* [59], and *Saccharum officinarum* [60,61], the aim of this article is to provide a survey of studies that have characterized the extracellular proteome of the model plant *Arabidopsis thaliana*. Further information regarding the cell wall proteome of the various aforementioned plant species is provided in the *WallProtDB* database, which accrues cell wall proteomic data, thereby facilitating the interpretation of said data as well as comparisons between cell wall proteomes [62]. This review will focus on various techniques employed to extract and enrich CWPs, including recent developments in protein separation methods that could be applied to future studies. Current challenges and the need for future targeted CWP proteomic studies are addressed. Finally, the role of CWP PTMs, as well as their contribution in response to biotic and abiotic stressors, is discussed.

## 2. Extraction and Enrichment of the Extracellular Proteome

**Figure 1:**
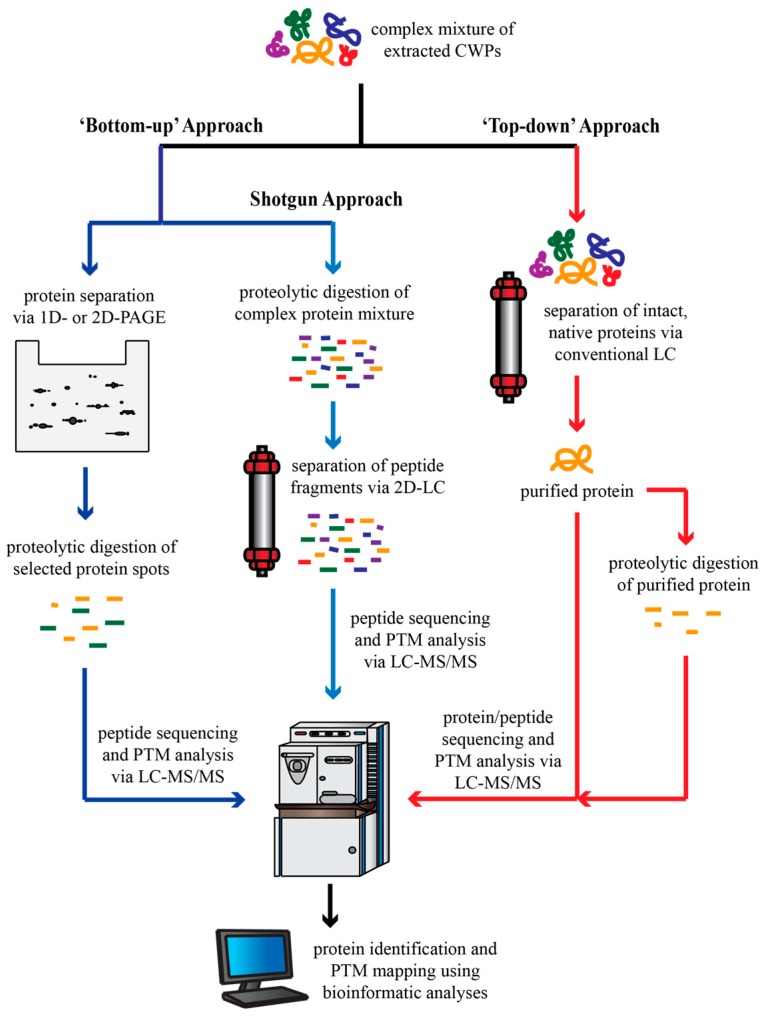
A schematic comparing the “top-down” and “bottom-up” approaches used in proteomic studies. CWP, cell wall protein; 1D, one dimensional; 2D, two dimensional; LC, liquid chromatography; MS/MS, tandem mass spectrometry; PTM, post-translational modification.

This review focuses upon the various methods employed to extract, enrich, and characterize the extracellular proteome of the model plant *Arabidopsis thaliana*. The particular focus is shared between the secretome, being all secreted proteins collected from a cell or seedling culture medium, and the cell wall proteome, which includes proteins residing in the apoplastic fluid as well as proteins existing within the cell wall polysaccharide matrix. In large-scale proteomic studies, the extracellular proteins are first extracted; the particular methods utilized for this initial step can vary widely, often depending upon the starting plant material and the proteins one is interested in recovering (i.e., loosely-bound versus tightly-bound CWPs). Once recovered, two very different approaches can be used to lead to the identification of the extracted CWPs (Figure 1). The “top-down” approach involves the isolation of intact, purified CWPs that retain their native conformation, and thus their biological activity. Conversely, the more commonly-used “bottom-up” approach involves the digestion of proteins into small peptides with an exogenous endopeptidase (typically trypsin), followed by the separation and identification of the resulting peptide fragments via mass spectrometry (MS). Traditionally, proteins were first separated using 2D-PAGE, with the resulting protein spots being picked and digested into peptide fragments that are then analyzed (e.g., sequenced) by MS to reveal the identity of the protein using bioinformatics databases (Figure 1). More recently, however, the shotgun style of “bottom-up” proteomics has become popular; this method involves the proteolytic digestion of total CWP extracts followed by multiple dimensions of peptide fragment separation, typically involving orthogonal liquid chromatography techniques (Section 3.1) (Figure 1) [63]. Protein identification routinely involves MS (Section 2.1), and bioinformatics analysis. After accounting for potential contamination by intracellular proteins, the collection of identified CWPs is considered to be the extracellular proteome. A summary of the reviewed literature can be found in Table 1.

### 2.1. The Secretome

The secretome encompasses the collection of proteins that are freely secreted into the liquid media in which suspension cells, roots, or seedlings are cultured. The secretome can also include “labile proteins”, being CWPs that have little or no interaction with other cell wall components, allowing them to move freely within the extracellular space and thus into the culture media. Suspension cell cultures have traditionally been the preferred choice for secretome studies because it is easy to separate the cells from the culture filtrate, as well as to check for damaged or dead cells as a proxy for estimating potential intracellular contamination [32]. However, it has been noted that suspension cell cultures do not reflect the natural environment, and that organ- and/or developmental-specific secretomes cannot be derived from this system [5,32,33]. As a result, interest is expanding away from utilizing cell cultures to other systems that reflect more natural growth conditions or comparing the secretome of cells under varying environmental situations, such as nutrient [68] or pathogen [69] stress.

**Figure 2:**
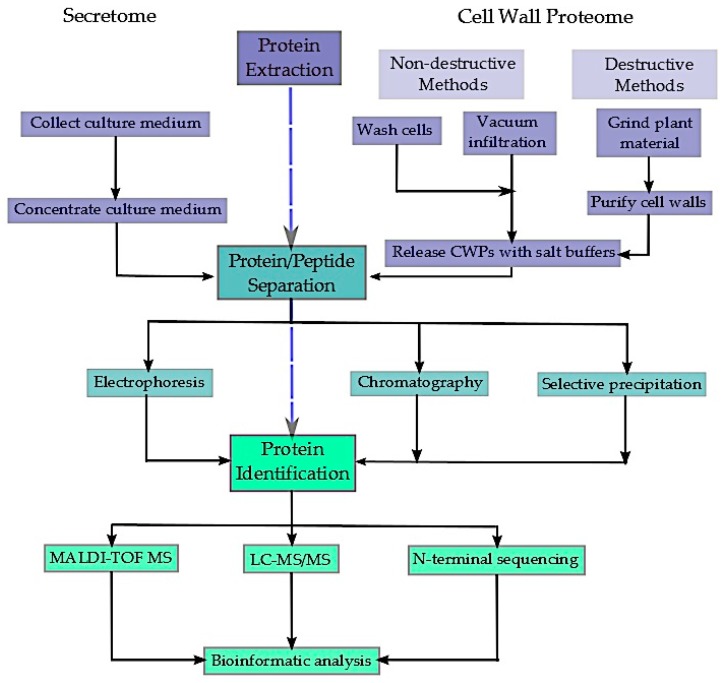
Summary of the sequence of steps employed in “bottom-up” proteomics to obtain the *Arabidopsis thaliana* extracellular proteome, including various methods of protein extraction, separation, and identification.

In contrast to the complexity of extracting proteins from the cell wall proteome, collecting the proteins belonging to the secretome is quite simple (Figure 2). Generally, the culture medium is concentrated and dialyzed. Concentrating can be done using various methods, including centrifugal filters [64,68], tangential flow ultrafiltration systems [66,68], freeze-drying [65,68], and acetone or methanol precipitation [67,69]. Traditionally, proteins are separated using one-dimensional (1D) [69] or 2D-PAGE [64,65,66,68], with protein-staining spots then being digested and identified by means of peptide sequencing via liquid chromatography-tandem MS (LC-MS/MS) [66,69], peptide mass fingerprinting via matrix-assisted laser desorption ionization time of flight MS (MALDI-TOF MS) [65,68], and/or multidimensional protein identification technology (MudPIT) analysis [66]. Lastly, bioinformatics analysis using Internet databases is used to identify the proteins, as well as determine whether any PTMs are present, including the potential cleavage of a secretion signal peptide. As proteomic, MS, and bioinformatics technologies continue to evolve, improved methods of proteome separation, analysis, and identification have become available (Section 3.1), allowing for the possibility of greater proteome coverage.

While the above methodology describes the basic sequence of events when identifying secretome proteins, additional steps can be undertaken to improve protein yield. In one case, polyvinyl(polypyrrolidone) was added to the culture filtrate of *Arabidopsis* cell cultures [64]. Polyvinyl(polypyrrolidone) acts to trap phenolic compounds, which helps to prevent their unspecific interaction with secreted proteins; such interactions can often result in the protein becoming more susceptible to aggregation, and thus precipitation [64]. The addition of polyvinyl(polypyrrolidone) to the culture filtrate facilitated the identification of proteins not previously found in earlier studies [64], suggesting that its inclusion improves protein yield and thus overall proteome coverage.

Lastly, it is important to consider intracellular contamination when isolating the extracellular proteome. When the filtrate of liquid-cultured seedlings was treated with neutral red, a stain that targets cell vacuoles, the stain was able to permeate through the intact seedlings into the cotyledons, hypocotyls, and roots [64]. This suggests that the culture medium has the potential to wash out proteins from intracellular spaces, indicating potential contamination. Consequently, the purity of the secretome is often analyzed by testing for the presence of relatively abundant cytoplasmic marker enzymes, such as malate dehydrogenase, aldolase, phosphoenolpyruvate carboxylase, glucose-6-phosphate dehydrogenase, or ribulose-1,5-bisphosphate carboxylase/oxygenase using immunoblotting with specific antibodies and/or enzyme activity assays [32,65,67,68]. Alternatively, if using suspension cell cultures, microscopy can reveal dead or damaged cells that might contribute to the contamination of the secretome by intracellular proteins.

### 2.2. The Cell Wall Proteome

The cell wall proteome extends beyond the proteins that are spontaneously released into the growth media to encompass proteins that exist within the apoplast, where they may or may not interact with other cell wall components such as polysaccharides. In order to release these proteins, either non-destructive or destructive methods can be used. In both cases, it is common to apply salt solutions in order to disrupt the non-covalent interactions between CWPs and cell wall polysaccharides [8,9,25,26,72,73,74,75,76,79]. CWPs can be differentially solubilized depending on what chemical is used for the extraction. Salts such as NaCl and CaCl_2_ are thought to extract CWPs retained by ionic interactions within the cell wall [70], although CaCl_2_ has proven to be more effective in releasing the greatest number of CWPs [72]. In contrast, NaCl and LiCl preferentially elute proteins containing interaction domains, such as hydroxyproline-rich glycoproteins [70,72]. Chaotropic agents, including urea, and chelating agents, such as EDTA or CDTA, can be included in the extraction buffer to disrupt hydrogen bonds and solubilize Ca^2+^-pectin complexes, respectively [6,70,72,79]. To disrupt strong interactions, such as cysteine disulfide bonds, detergents such as SDS or Triton and sulfhydryl reducing agents such as dithiothreitol can be utilized [8,79]. In some cases, the sequential use of contrasting salt solutions has been employed to maximize the number of proteins being extracted [6,9,70,72,74,75,79]; however, using too many washes of salt solutions can increase the risk of contamination by intracellular proteins [70].

Non-destructive methods aim to release CWPs while preserving the integrity of the plasma membrane so as to minimize the potential for intracellular contamination. These methods can range from simply washing suspension cells in the extraction buffer of choice [25,68,70,79] to using vacuum infiltration to introduce the extraction buffer into the apoplast of seedlings or leaves, followed by the collection of the buffer and extracted CWPs via centrifugation [71,72,73]. Osmotic agents, such as mannitol, have been used to plasmolyze cells prior to extraction, increasing the physical space between the cell wall and plasma membrane, thereby reducing the risk of contamination of the extracellular proteome by intracellular proteins [25,70,72]. Despite the non-destructive nature of these methods, damage can still occur to the cells. In particular, using CaCl_2_ to wash suspension cells may increase the risk of intracellular contamination [70].

Destructive extraction methods involve homogenizing the tissue before extraction, thereby mixing the extracellular and intracellular fractions. Consequently, this extraction method must be followed by further steps to separate the extracellular and intracellular proteomes. Cell walls can be purified from the ground tissue via sedimentation in glycerol [6,8,9,26,74,75]. These purified cell wall fragments can then be extracted using salt solutions to retrieve the CWPs. Alternatively, the tissue may be ground in the presence of salt solutions, leaving CWPs and intracellular proteins together in the homogenate. In this case, the homogenate is then enriched for specific CWPs using methods that depend upon the characteristics of the proteins of interest. Lectin affinity columns can successfully enrich N-linked glycoproteins [75,76]. Concanavalin A is the most common, and reportedly the most effective, lectin affinity column; however, wheat germ agglutinin, peanut agglutinin, *Sombucus negra* lectin, and *Artocarpus integrifolia* lectin have also been used [1,75,76]. Arabinogalactan proteins can be selectively precipitated using the β-glucosyl Yariv reagent [78]. Finally, a phosphatidylinositol-specific phospholipase C has been used to isolate glycosylphosphatidylinositol-anchored proteins [77].

Once the CWPs have been extracted, whether by non-destructive or destructive means, they must be separated and identified. Traditionally, 2D-PAGE has been the most popular method of protein separation [25,26,68,72,76,80], despite the challenges related to: (i) CWPs being largely basic, making separation based on isoelectric point ineffective; and (ii) the difficulty of 2D-PAGE to detect low abundance and/or high molecular weight (i.e., >100 kDa) CWPs as well as very low molecular weight, extracellular signalling peptides such as systemin. To overcome these challenges, other methods of protein separation are being increasingly utilized. These include difference gel electrophoresis (DIGE) [73,77], MudPIT analysis [8], and liquid chromatography [74,78]. Peptide sequencing and analysis via LC-MS/MS under the shotgun approach is the preferred choice for protein identification and PTM analysis, although peptide mass fingerprinting via MALDI-TOF MS and N-terminal microsequencing via Edman degradation have also been used [8,9,25,26,71,72,73,74,75,76,77]. As for the secretome, the purity of the CWP extract is assessed via assays of cytoplasmic marker enzymes, with glucose-6-phosphate dehydrogenase being most commonly utilized [25,71,73]. Microscopy is also an effective tool for inspection of extracts prepared by non-destructive methods, as any damage to the plasma membrane could indicate potential intracellular contamination [25].

**Figure 3:**
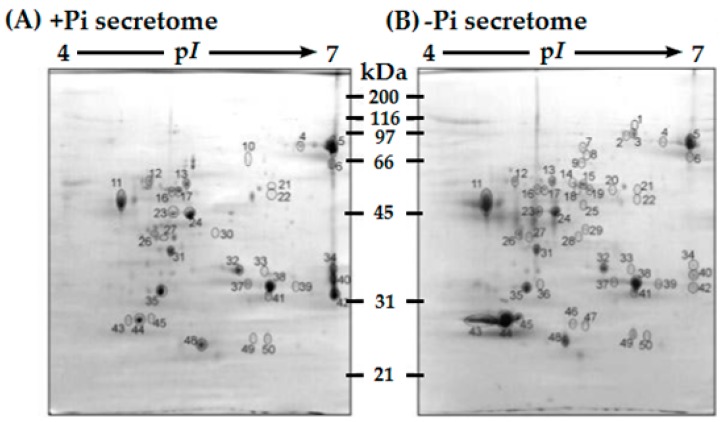
2D-PAGE comparison of protein-stained secretomes collected from seven-day old: (**A**) fully Pi-sufficient (+Pi); and (**B**) Pi-starved (−Pi) *Arabidopsis thaliana* suspension cell cultures (modified from [68]). When studying and the secretome or cell wall proteome, it is imperative to consider the macronutrient status of the culture media, as it can greatly impact the protein profile obtained.

A further concern that can markedly influence the extracellular proteome obtained from liquid-cultured cells or seedlings is the macronutrient status of the media itself. In particular, the 1.25 mM phosphate (Pi) concentration of conventional Murashige–Skoog media has been reported to be inadequate for maintaining batch-cultured suspension cells of various species in fully Pi-sufficient conditions for more than a couple of days post-subculture [68,81,82]. For example, seven days following subculture of *Arabidopsis* suspension cells into conventional Murashige–Skoog media: (i) Pi was undetectable in the cell culture media; and (ii) biomass accumulation was reduced by at least 25% relative to cells that had been initially subcultured in Murashige–Skoog media containing 5 mM Pi [68]. Moreover, the 2D-PAGE protein spot profile of concentrated secretome harvested from media of fully Pi-sufficient seven-day-old *Arabidopsis* suspension cells (i.e., that were subcultured at day-0 in media containing 5 mM Pi) was remarkably different that obtained from seven-day-old Pi-deprived cells (Figure 3). Many studies involve harvesting the culture filtrate or *Arabidopsis* cells or seedlings 5–7 days after sub-culturing into conventional Murashige–Skoog media [6,8,25,26,69,72,76]. Thus, these proteomic studies may have been inadvertently performed with nutrient-stressed cells, thereby altering the extracellular proteome that was obtained. Liquid Murashige–Skoog media needs to be supplemented with additional Pi, or be resupplied with Pi at regular intervals, in order to avoid inducing a Pi starvation response several days later [68].

## 3. Overcoming the Challenges of Identifying the Extracellular Proteome

As mentioned previously, there are numerous challenges related to the elucidation of the extracellular proteome, including protein separation and non-classically secreted proteins. Here, the focus will be on emerging methods that may be used to overcome these challenges, thus allowing a more comprehensive understanding of the extracellular proteome to come to light.

### 3.1. Advances in Protein Separation Techniques

Within the field of proteomics, no one method is able to, in a single step, identify and quantify the complete set of proteins derived from a complex sample due to difficulties surrounding the resolution of the various proteins [83]. 2D-PAGE has been the most common technique utilized for global differential proteomic studies, typically performed with intact proteins. However, 2D-PAGE has relatively low resolution, with multiple proteins making up a spot and/or the same protein being represented by multiple spots due to PTMs [83]. To improve the resolution of proteins, a modified 2D-PAGE system, known as difference gel electrophoresis has been developed. This system fluorescently tags protein samples (e.g., from a control versus “stressed” cell culture or seedling tissue) with different cyanine-based dyes before electrophoresis. This allows for the same multiplexed 2D-PAGE gel to be run with proteins obtained from the different samples that have been respectively tagged with different dyes, ultimately providing better detection of proteome differences between two protein samples within the same gel [83]. While large-scale “bottom-up” proteomic studies have traditionally made use of 2D-PAGE as a method of protein separation, the shotgun approach of multidimensional peptide fragment separation has begun to eclipse it. The proteolytic degradation of crude protein extracts results in highly complex samples that require the combination of multiple, orthogonal separation procedures to resolve all of the components [63,83]. As such, multidimensional chromatography methods have been employed to reduce sample complexity and to maximize the resolution and dynamic range, thereby increasing proteome coverage [63]. The majority of 2D-LC analyses utilize strong cation exchange (SCX) chromatography for the first dimension, followed by reverse-phase (RP) chromatography in the second dimension due to the high degree of orthogonality between the two columns and the compatibility of RP chromatography with electrospray ionization (ESI) mass spectrometry [63]. Such is the case with MudPIT analysis, which combines a SCX and RP column in one fused capillary needle that also acts as an ESI emitter [8,63]. Although SCX and RP columns are most commonly used, other strategies include affinity purification chromatography, size exclusion chromatography, various combinations of ion exchange columns, or even anion and cation mixed-bed exchange columns; these strategies can be used in multiple combinations to create high orthogonality between individual dimensions and thus provide enhanced separation of peptide fragments, increasing the peak capacity of the overall approach [63,83]. Not only can orthogonal, multidimensional separation techniques be used to improve proteome coverage, but they can also be employed to enrich peptide fragments containing a PTM of interest. For example, under low pH conditions, the combination of SCX chromatography followed by weak anion exchange chromatography can enrich phosphorylated peptide fragments [63]. Hydrophilic interaction chromatography (HILIC) may aid in enriching peptides having polar PTMs (e.g., phosphorylation and glycosylation), particularly when combined with immobilized metal affinity chromatography (IMAC) and zwitterionic-HILIC, respectively [63]. Likewise, electrostatic repulsion-HILIC and SCX-IMAC can be used in a complementary fashion to recover a comprehensive phosphoproteome [63,84]. Proteins and their respective PTMs can also be quantified using chemical-labelling techniques including dimethyl labelling, tandem mass tag (TMT), isobaric tags for relative and absolute quantification (iTRAQ), and stable isotope labelling by amino acids in cell culture (SILAC), further clarifying the emerging picture of plant CWPs and their regulation [84]. Alternatively, label-free quantification is becoming popular, having recently been employed to assess the relative quantity of proteins localized to the *Arabidopsis* Golgi apparatus and chloroplast stroma using ion mobility separation, known as LC-IMS-MS^E^ or HDMS^E^, whereby ion current is recorded for a peptide ion and is used as a measure of its abundance [85,86]. As the technology continues to develop, both the resolution and coverage of CWPs will improve in kind, untangling the complexity of the cell wall proteome.

### 3.2. The Advantages of Targeted Proteomics

**Figure 4:**
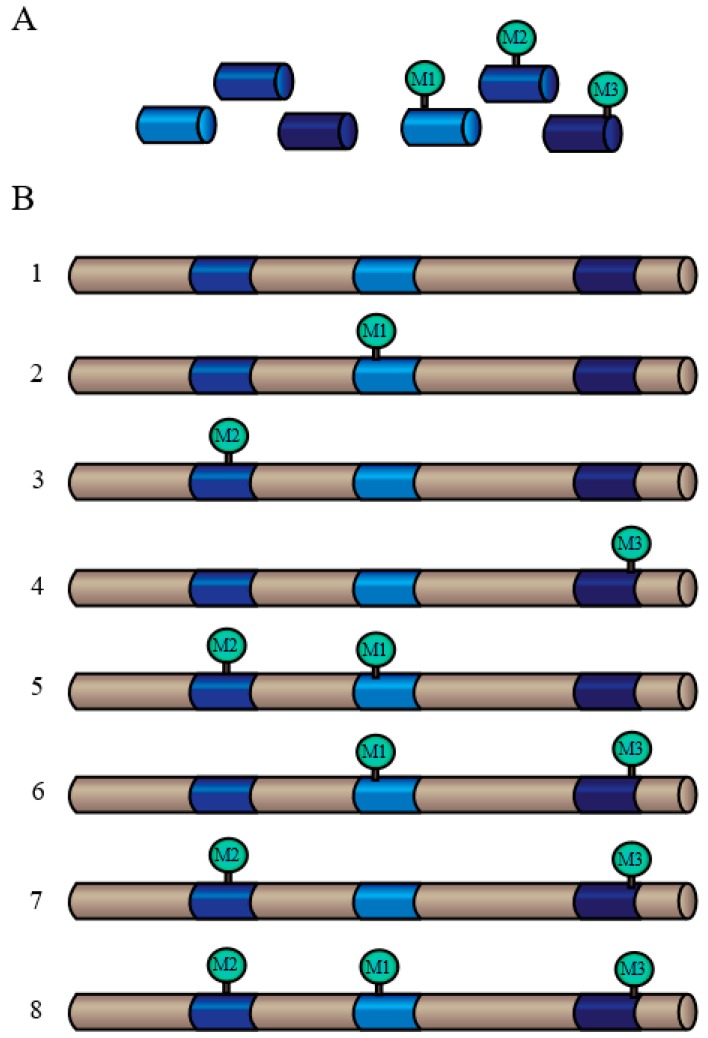
The “bottom-up” proteomic approach cannot resolve multiple proteoforms that may exist simultaneously in vivo: (**A**) three peptide fragments derived from the same protein may carry a PTM (M1, M2, M3) or exist unmodified; and (**B**) all eight proteoforms of the same protein could generate the peptide fragments found in (**A**). Figure modified from [84].

As mentioned previously, the traditionally-used 2D-PAGE method of protein separation results in fairly low resolution and inherently biases high-abundance and lower molecular weight proteins, as they would be more readily separated and visualized on the gel. Consequently, many important CWPs can be overlooked, despite other evidence of their presence in the extracellular proteome. Exemplifying this point is a pair of Pi-starvation inducible *Arabidopsis* purple acid phosphatase (PAP) isozymes, AtPAP12 and AtPAP26 (*At2g27190* and *At5g34850*, respectively). When *Arabidopsis* suspension cells were cultured in Pi deficient Murashige–Skoog media, a significant increase in secreted acid phosphatase (APase) activity was measured, due to the upregulation of these major secreted PAP isozymes [87]. Despite this upregulation, their protein-staining spots were not visible following 2D-PAGE of the corresponding secretome, owing to their low abundance in the culture media relative to other secreted proteins. As such, a targeted, “top-down” proteomics approach was undertaken to characterize the secreted APases involved in Arabidopsis Pi scavenging and acquisition. The culture filtrate of seven-day old Pi-starved suspension cells was concentrated and native APases were purified using a series of chromatography columns to yield homogenous and active AtPAP12 and AtPAP26 that could be readily identified via MS and N-terminal microsequencing [87]; AtPAP12 and AtPAP26 were unequivocally identified using this targeted approach and subsequently shown to play pivotal roles in the Pi-acquisition efficiency of Pi-starved *Arabidopsis* seedlings [88]. Furthermore, these active PAP preparations were not only suitable for detailed studies of their respective kinetic properties, but also for identifying any PTMs that may be present. In this case, targeted proteomics led to the discovery of multiple glycoforms of AtPAP26, each having distinctive kinetic properties [87,89]. This becomes particularly relevant considering the shortcomings of the shotgun method with respect to determining the presence and/or site of a PTM. In this approach, the hydrolysis of peptide bonds is done using proteolytic enzymes, most commonly trypsin; however, many peptide fragments produced by trypsin may be very small and thus not easily identified by MS, resulting in a restricted segment of the proteome being covered [90]. Moreover, negatively-charged amino acids, such as aspartic or glutamic acid, or phosphorylated serine or threonine residues are often not cleaved by trypsin when located in close proximity to an arginine or lysine residue [90]. These missed cleavages result in longer peptide fragments with higher charge states from the internal arginine or lysine residues. These longer peptide fragments are unsuitable for higher-energy collisional dissociation (HCD) that is commonly used for phosphosite assignment via MS [90]. As such, many potential PTMs may be overlooked based solely on the fragmentation of the protein. The “bottom-up” approach is also unable to provide an exact determination of proteins with multiple PTM sites, as examining several peptide fragments of the same protein, each of which may or may not be modified, cannot resolve the various potential proteoforms that may exist in vivo (Figure 4) [84]. As illustrated by studies of secreted AtPAP12 and AtPAP26 in Pi-starved *Arabidopsis*, the analysis of intact proteins via the “top-down” proteomics approach can overcome some of the challenges faced by the shotgun approach, including identifying low-abundance CWPs and definitively characterizing any PTMs with high sequence coverage. As mentioned, this targeted method also yields active, native enzymes that can be employed for detailed kinetic and protein interaction studies. Currently, approximately 90% of CWPs have been categorized based upon predicted biochemical or biological function [3]. Using targeted proteomic analysis, the biochemical function of identified CWPs could be experimentally verified, providing more confidence when organizing proteins into functional categories. This may be of particular interest since many CWPs could be “moonlighting” enzymes that have multiple biological functions and/or catalytic activities. For example, purified AtPAP26 also displays alkaline peroxidase activity in addition to APase activity, suggesting that this enzyme may also be involved in the metabolism of extracellular reactive oxygen species [89].

While large-scale, peptide-based proteomic studies have provided a wealth of information regarding CWPs, it is apparent that a more narrow, “top-down” approach is required to gain a complete understanding of which proteins make up the extracellular proteome, and what roles they play in that space. Utilizing traditional protein purification techniques such as ion-exchange, hydrophobic interaction, gel filtration, and affinity chromatography paves the way for more targeted separation of native, biologically active CWPs. Such targeted analysis could allow for the identification of CWPs previously overlooked by 2D-PAGE techniques, the identification of important PTMs, and the eventual elucidation of their definitive biological and biochemical roles.

### 3.3. Confirming Protein Localization

Many methods have been developed to extract the extracellular proteome, or a subset thereof. However, the issue of non-canonical CWPs continues to confound these efforts. Fortunately, continual advances in molecular biology make it feasible to screen large cDNA libraries to confirm protein localizations through fluorescent or colorimetric visualization [21,91,92,93,94,95,96]. Fluorescent tagging of full-length proteins can be used to follow the localization of selected proteins. Genes encoding putative CWPs can be fluorescently tagged at an internal site so as to preserve the native gene regulatory sequence; these tagged proteins can then be stably expressed in transgenic *Arabidopsis* plants where the protein product can be visualized via fluorescence microscopy [91]. In a proof-of-concept experiment, the *Arabidopsis* proline-rich protein 2 (*At2g21140*), a known CWP, was fluorescently tagged and later visualized within the cell wall, as expected [91]. This method may then be extended to test many potential CWPs so as to provide confirmation of their extracellular localization. A second visualization technique involves secretion trap assays. The yeast secretion trap assay [93,95] fuses the cDNA of interest to the 5’ end of a mutant yeast invertase gene that lacks a secretion signal peptide. This fusion can then be transformed into a *suc2* mutant of *Saccharomyces cerevisiae* and be plated on a sucrose selection medium. Invertase catalyzes the hydrolysis of sucrose into fructose and glucose. Since the *suc2* yeast mutant cannot utilize sucrose as a carbon source, it requires the secretion of invertase into the sucrose medium as a requisite to grow. However, invertase will only be secreted into the medium if the cDNA of interest encodes a secreted protein. As such, all yeast transformants that contain a cDNA encoding a secreted protein should be able to rescue the mutant, thereby providing an effective screen to isolate CWPs. A second secretion trap can be done within the *Arabidopsis* plant itself [94]. This method utilizes a modified Ds transposable element that carries the β-glucuronidase (GUS) reporter gene, and assumes that secreted proteins are glycosylated. Gene trap insertion lines were generated by mobilizing the transposable element, resulting in a library of unique, stabilized transposon insertion sites. The insertion lines could then be grown, either with or without tunicamycin, and the GUS-staining activity could be compared between these treatment groups. The GUS fusion proteins routed through the secretory pathway are enzymatically inhibited by N-linked glycosylation, which results in a decrease in colorimetric staining; in the presence of tunicamycin, glycosylation is inhibited, releasing the repression of GUS activity. Therefore, for a secreted protein, one would expect to see no staining under the control treatment, when glycosylation can occur, and see staining present when seedlings are treated with tunicamycin. *Arabidopsis* seedlings demonstrating this pattern of colorimetric staining likely have the transposable element inserted within a gene encoding a secreted protein.

These visualization techniques could aid in confirming the localization of proposed CWPs and contribute to the differentiation between intracellular contaminants and non-classically secreted proteins, thus helping to overcome one of the many hurdles involved in defining the extracellular proteome.

## 4. Post-translational Modifications of Cell Wall Proteins

Cell wall proteins are involved in many processes related to cell wall assembly, remodelling, and cell-to-cell signalling in response to environmental cues [22]; directing these activities are PTMs. Newly synthesized proteins are generally inactive in their native form, and often undergo a number of modifications to become a mature, functional protein [97]. PTMs can modulate protein interaction, localization, and the stability of the modified protein, and so play an integral role in protein regulation. The majority of proteins require at least one PTM to confer activity and stability [97,98]. Currently, more than 300 types of protein PTMs have been characterized, with new cases continually being discovered [99]. The most important PTMs of eukaryotic proteins include glycosylation, phosphorylation, attachment of fatty acids (i.e., glypiation, myristoylation, prenylation, etc.), and proteolysis [97,98]. Many global analyses of extracellular proteomes have reported that different spots on a 2D-PAGE gel represent the same gene product, indicating that PTMs are present and that they have an influence on the isoelectric point and/or the molecular weight of the affected CWPs [25,26,65]. Traditionally, Edman degradation, amino acid analysis, isotopic labelling, and immunochemistry have been used to identify protein PTMs; however, since the covalent modification of a protein alters its molecular weight, and thus the mass of the modified amino acid, MS has become the more popular technique utilized for the identification and characterization of proteins and their associated PTMs [97,98]. While it is relatively simple to identify a protein using minimal sequence coverage of one or two peptides, pinpointing and characterizing all of its PTMs, as well as how they change during development or following exposure to various stresses, requires nearly 100% sequence coverage [97,98]. Attaining maximal sequence coverage normally requires digesting the protein with several different proteases, such as trypsin, chymotrypsin, and endoproteinase Asp-N, and then analyzing each peptide digest via LC MS/MS [99]. In this section, several of the most important PTMs of CWPs are discussed.

### 4.1. Glycosylation

Glycosylation refers to the covalent attachment of an oligosaccharide side chain to a protein, representing the most complex PTM that a protein can undergo [23]. Glycosylation can directly influence the physical and chemical properties of the affected protein, including its thermal resistance, solubility, proteolytic degradation, specific activity, and interactions with ligands and/or other proteins [97]. The various flavours of glycosylation have been extensively reviewed in previous articles [23,97]. This review, however, will focus on the most common forms of glycosylation as related to CWPs: *N-*glycosylation and *O-*glycosylation.

**Figure 5:**
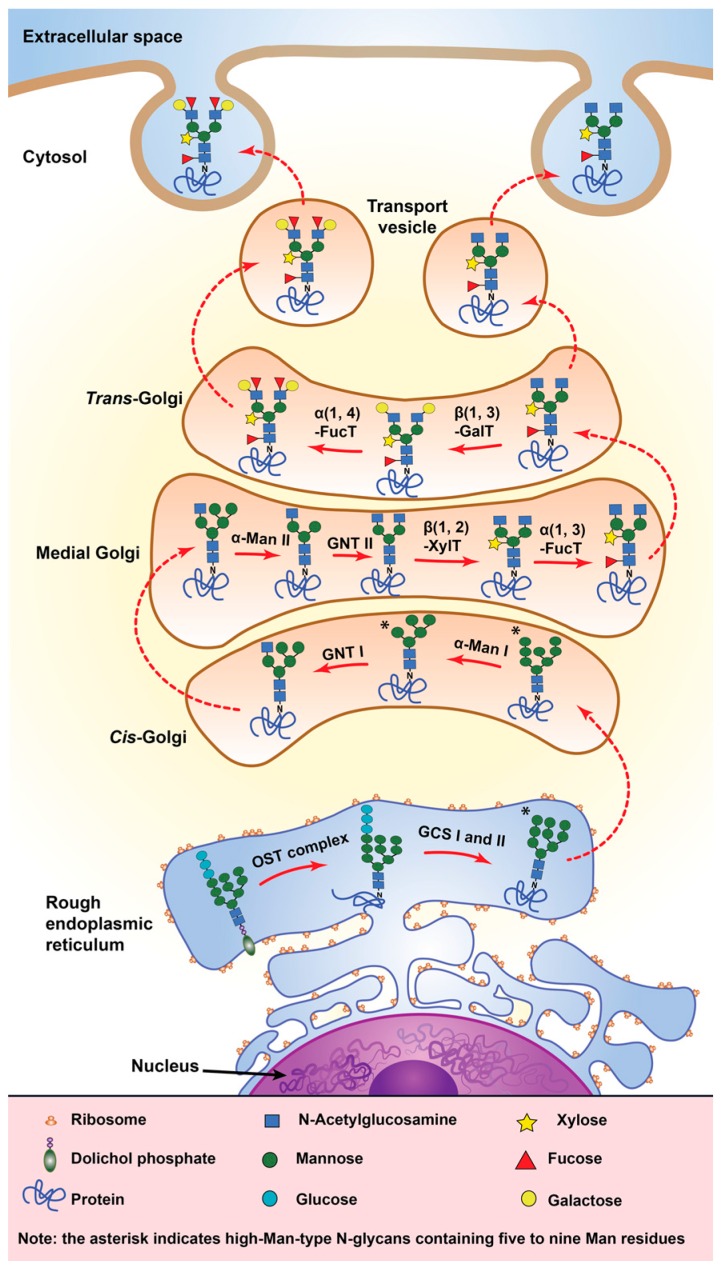
Protein *N*-glycosylation pathway in plant cells. The *N*-glycosylation of proteins begins with the transfer of a dolichol lipid-linked oligosaccharide precursor, Glc_3_Man_9_GlcNAc_2_, to the Asn residue of the nascent protein by the activity of oligosaccharyltransferase complex (OST complex) within the endoplasmic reticulum. The newly formed *N*-glycoprotein undergoes a trimming of the three glucose units by the activity of glucosidases I and II (GCS I and II). The *N*-glycan is then further modified in the Golgi apparatus. In the *cis*-Golgi, α-mannosidase I (α-Man I) removes four mannose residues, followed by the addition of an initial GlcNAc residue to the α(1,3)-mannose branch catalyzed by *N*-acetylglucosaminyltransferase I (GNT I). In the medial Golgi, α-mannosidase II (α-Man II) removes two more mannose residues and the second GlcNAc residue is added to the α(1,6)-mannose branch by the activity of *N*-acetylglucosaminyltransferase II (GNT II). This step is followed by α(1,3)-fucosylation and β(1,2)-xylosylation actions catalyzed by α(1,3)-fucosyltransferase (α(1,3)-FucT) and β(1,2)-xylosyltransferase (β(1,2)-XylT), respectively. The complex-type *N*-glycan can be further modified by the addition of terminal fucose and galactose residues within the *trans*-Golgi. A β(1,3)-galactosyltransferase (β(1,3)-GalT) and an α(1,4)-fucosyltransferase (α(1,4)-FucT) are responsible for the final addition of galactose and fucose residues, respectively.

*N-*glycosylation is the most common carbohydrate-peptide bond found within plant cells [23]. It involves the β-glycosylamine linkage of *N*-acetylglucosamine (GlcNAc) to the amide nitrogen of an asparagine residue occurring in the consensus sequence Asn-X-(Ser/Thr) by the oligosaccharyltransferase enzyme complex, where X can be any amino acid except proline [23]. In some exceptional cases, such as for sweet corn amylogenin, arginine is the target *N-*glycosylation site [23]. *N-*glycosylation is initiated during protein translation in the endoplasmic reticulum with the transfer of a precursor oligosaccharide Glc_3_Man_9_GlcNAc_2_ from a dolichol lipid carrier to the specified Asn residues of the nascent growing polypeptide (Figure 5). As the newly-translated glycoprotein transfers from the endoplasmic reticulum to the Golgi apparatus, the *N-*linked polysaccharide undergoes several iterations of trimming and additions of sugar residues by glycosidases and glycosyltransferases to become a fully mature *N-*linked glycan [100,101,102]. These maturation steps can result in the production of mannose-type *N-*glycans and complex-type *N-*glycans, which are characterized by having an α(1,3)-fucose and/or a β(1,2)-xylose residue attached to the proximal glucosamine residue and to the β-mannose, respectively [97,101,102].

In contrast to *N-*glycosylation, *O-*glycosylation involves the attachment of the carbohydrate chain to the hydroxyl end of threonine, serine, or hydroxyproline residues, although tyrosine and hydroxylysine have also been reported as potential target residues for *O-*glycan linkage in non-plant cells [97]. *O-*glycosylation also differs from *N-*glycosylation in anomeric configuration; both α- and β-forms are found for *O-*glycosidic bonds, whereas *N-*glycosidic bonds are only known to exist in the β-form. Glycoproteins can themselves exist in several different glycoforms that contain distinct oligosaccharide chains attached to specific glycosylation sites. The heterogeneous glycosylation pattern of glycoforms can result in significant modulation of the activity, subcellular location, protein interactions, and biological function of the target glycoprotein. Such is the case for a pair of differentially glycosylated, Pi-starvation inducible AtPAP26 glycoforms that were fully purified from the cell wall (AtPAP26-CW1 and -CW2) and secretome (AtPAP26-S1 and -S2) of Pi-deprived *Arabidopsis* suspension cells [87,103]. A 55 kDa protein exclusively copurified with the AtPAP26-CW2 and AtPAP26-S2 glycoforms, and was identified via MS as a curculin-like lectin [104]. Lectins are carbohydrate-binding proteins that interact with the glycan groups of glycoproteins [105,106]. Bifluorescence complementation, Far Western immunoblotting, and glycoprofile analysis by LC MS/MS all indicate that AtPAP26-CW2 and -S2 specifically associate with the curculin-like lectin, and that this interaction (which appears to stimulate their APase activity) is mediated by the unique oligosaccharide groups found on these glycoforms, highlighting the important role of glycosylation in determining a protein’s properties and function [104].

Glycosylation is, by far, the most ubiquitous PTM found within the cell wall [98,107]. The majority of proteins associated with the cell wall are expected to be *N*-glycosylated, as they pass through the secretory pathway to reach the extracellular environment. Only a handful of CWPs have been detected with no *N-*glycosylation site, such as expansins, which are *O-*glycosylated [22]. The cell wall glycoproteome can be enriched using methods such as lectin affinity chromatography for *N*-linked glycoproteins or the β-Yariv reagent to selectively precipitate arabinogalactan proteins [1,75,76,78]. Alternatively, if using the shotgun approach, ZIC-HILIC can be employed to enrich glycosylated peptide fragments [63]. Such techniques have allowed for the identification, and downstream characterization, of glycosylated CWPs.

Hydroxyproline-rich glycoproteins (HRGPs) are the major surface glycoproteins in plants, associating with either the cell wall or plasma membrane, where they play an important role in determining cell shape, plant morphology, and cell wall strength [97,108]. Overexpression of prolyl 4-hydroxylases genes, which encode key enzymes involved in the glycosylation of HRGPs, increases root hair length and density [107]. This results in a greater overall plant biomass due to the significant increase in nutrient and water assimilation, demonstrating the importance of glycosylated CWPs in determining cell shape and morphology [107]. In addition, RSH (root-shoot-hypocotyl-defective), a member of the HRGP superfamily that is localized to the cell wall, is required for the correct positioning of the cell plate during cytokinesis and normal embryo development in carrot cells [108]. The loss of RSH results in abnormally shaped cells in developing carrot embryos, further emphasizing the critical roles that glycoproteins play in cell division, and therefore cell shape [108]. Likewise, in *xeg113* mutants, where extensins are abnormally arabinosylated, the termination of cell wall growth is delayed, resulting in elongated plants as compared to a wild type control [109].

When the extracellular proteome of regenerating *Arabidopsis* protoplasts were surveyed, the ratio of glycoproteins to total proteins remained relatively static (approximately 0.7) throughout the different stages of protoplast development, indicating that a specific level of glycoproteins are required for normal cell wall regeneration [25]. However, this study focused exclusively on the early stages of cell wall regeneration; the glycoprotein to protein ratio could change in the late stages of cell wall regeneration or during cell growth. Considering the critical roles that glycoproteins play in cell biology, further studies should be pursued to fully understand the roles of glycoproteins in the cell wall during cellular development and stress responses.

### 4.2. Phosphorylation

Reversible phosphorylation is the most predominant and well-studied PTM of eukaryotic proteins [14,15,25,73,104]. Greater than 70% of all eukaryotic proteins are believed to be phosphorylated at some point during their lifetime, with the majority of these proteins having multiple phosphorylation sites [104]. Phosphoproteomics refers to the study of varying aspects of protein phosphorylation, including phosphoprotein identification, quantification, mapping of phosphorylation sites [14]. In eukaryotes, protein phosphorylation occurs predominantly on serine and threonine residues, as well as less commonly on tyrosine residues [28,110,111]. The addition or removal of a phosphoryl group (PO_3_^2−^), catalyzed by protein kinases and phosphatases, respectively, alters the function and activity of proteins participating in virtually all aspects of cellular physiology and development including signal transduction, cell differentiation, disease and stress responses, and metabolic pathway flux [104,112]. Protein kinases are one of the largest gene super families in eukaryotes, with approximately 1100 different protein kinases encoded in the genome of *Arabidopsis*, illustrating the crucial role of protein phosphorylation in the control of diverse cellular processes [14,104,112]. These protein kinases can present diverse substrate specificities, due in part to the nature of the amino acid residues flanking the target phosphorylation site; such a large degree of variation makes it difficult to predict the position of biologically active sites for protein phosphorylation [113]. As such, detailed phosphoproteomic studies are necessary to understand when and where protein phosphorylation events take place.

While the general mechanism and functions of reversible protein phosphorylation are well understood, the associated signalling pathways and impact of phosphorylation on biological properties of specific targets, especially CWP phosphoproteins, are poorly defined. Phosphoamino acid-specific antibodies and/or Pro-Q Diamond phosphoprotein staining of 1D- or 2D-PAGE gels provides insights into protein phosphorylation events taking place within the cell wall or secretome [25,26,58]. Multidimensional chromatography setups, such as SCX-IMAC or HILIC-IMAC, may also identify phosphorylation events by enriching the phosphorylated peptide fragments produced during the shotgun proteomics approach [63,84]. When *Arabidopsis* suspension cells were treated with fungal elicitors or subjected to nutritional Pi deprivation, marked changes in the profile of the extracellular proteome occurred, including differential phosphorylation of certain CWPs [26]. These changes were hypothesized to be part of a signal transduction cascade mediating cellular responses to fungal infection or Pi deprivation. Additionally, alterations in phosphorylation status of many CWPs occurred during early protoplast regeneration of *Arabidopsis* [25]. Phosphorylated CWPs include expansins, α-xylosidase, β-xylosidase, β-galactosidase, α-mannosidase, phosphatases, phosphoesterases, isomerases, lectins, dehydrogenases, and an aspartyl protease [25]. Continued global analysis of the phosphorylation status of CWPs under varying environmental and developmental conditions, in-depth phosphoproteomic studies, and the occurrence and functions of secreted protein kinases [114] and apparent protein phosphatases [103], will be required to more completely elucidate the functions and mechanisms of reversible protein phosphorylation in the extracellular proteome. Identification of proteins whose phosphorylation status is significantly modulated during stress acclimation, or during recovery from stress, is just the “tip of the iceberg” since this information raises five important questions [104], as follows:

1. *Where* is/are the site(s) of phosphorylation? Mapping the specific amino-acid residue of a protein that has been phosphorylated in vivo provides fundamental information regarding the mechanistic details, as residues flanking the phosphorylation site often contain an essential targeting “motif” for recruitment of the responsible protein kinase isozyme.
2. *What* is the stoichiometry of in vivo phosphorylation? Phosphorylation stoichiometry must be significant in order for this PTM to have a meaningful impact on the biological function of the target protein in a living cell.
3. *When* is/are the particular residue(s) phosphorylated? Defining the timing and order of changes in the phosphorylation status of specific proteins following exposure to, and during recovery from, various stressors.
4. *How* is/are the particular residue(s) phosphorylated? Pinpointing a protein’s in vivo phosphorylation site can lead to identification and characterization of the responsible protein kinase, and ultimately to the upstream signal-transduction pathways that control its expression and/or activity
5. *Why* is the protein phosphorylated? This is perhaps the most compelling question of all, since even when Questions 1 to 4 are fully answered, we must ultimately understand how phosphorylation influences the activity and biological function(s) of the target protein.

### 4.3. Other PTMs

Ubiquitination describes the covalent attachment of the small protein ubiquitin to one or more lysine residues of a target protein through the action of three enzymes: the ubiquitin activing enzyme (E1), the ubiquitin conjugating enzyme (E2), and the ubiquitin protein ligase (E3) [104,115]. E1 activates ubiquitin by forming an energy-dependent thioester bond with the C-terminus of ubiquitin. The activated ubiquitin is then transferred from the E1 enzyme to the target cysteine residue on the E2 enzyme via a trans (thio)esterification reaction. In the final step of the ubiquitination cascade, E2 transfers ubiquitin directly to the E3 ligase, which then transfers ubiquitin to the target protein [115]. The specificity of ubiquitination is ensured by the large variety of E3 ligases, which each recognize particular target proteins. For example, approximately 1400 *Arabidopsis* genes are predicted to encode different E3 ligases [116,117]. Ubiquitination can occur as either polyubiquitination, a destructive PTM that tags proteins with multiple ubiquitin chains, resulting in their rapid elimination by the 26S proteasome, or as monoubiquitination, which involves the attachment of a single ubiquitin protein to the target, leading to some sort of reversible regulatory effect [104,117,118]. Monoubiquitination can regulate a diverse number of proteins, including histones, endocytic machinery, transcription factors, and metabolic enzymes by altering their activity, interactions with other proteins, and/or localization [119,120,121,122]. In yeast and mammalian cells, monoubiquitination can act as a sorting signal within the secretory/endocytic pathway [121,122]. This function may be conserved across all eukaryotes, perhaps contributing to the localization of CWPs. Polyubiquitination, and by extension proteolysis, can also play an important regulatory role within the cell wall. Treatment of *Picea wilsonii* with MG132, a proteasome inhibitor, interrupts the ubiquitin/proteasome pathway that is involved in the degradation of misfolded proteins, as well as short- and long-lived regulatory proteins, resulting in significant decreases in major pollen tube CWPs including pectin and cellulose [123].

Besides the degradation of full proteins by the 26S proteasome, proteolysis can include other irreversible cleavage processes by which a protease breaks down a protein into a smaller polypeptide via the removal of a signal peptide or propeptide, thus conferring new or modified activity [97,124]. CWPs that follow the classical secretion pathway generally contain a cleavable signal peptide that directs them to the cell wall [8]. Pectin methylesterases (PMEs) regulate pectin properties through the demethylesterification of cell wall polygalacturonans, which represent a major class of pectins. *Arabidopsis* PMEs include more than sixty isoforms, and are tightly controlled at the post-translational level. Type I PMEs require proteolysis to release the C-terminal PME domain from the pro region as a prerequisite to secretion of the protein into the cell wall [125]. Without proteolytic cleavage, the protein cannot reach its proper destination.

It becomes clear that identifying and characterizing the PTMs of CWPs is critical to understanding the dynamic of the extracellular proteome. Current bioinformatic programs allow for the prediction of some PTMs; however, these predictions are not always accurate and need to be verified at the experimental level using techniques such as LC-MS/MS of the native, purified protein. For example, SignalP (http://www.cbs.dtu.dk/services/SignalP/) predicted that the N-terminus of the deduced AtPAP26 polypeptide (a CWP upregulated by Pi-deprived *Arabidopsis*) contains a 21 amino acid signal peptide [89]. However, an actual signal peptide of 29 amino acids was revealed by comparing the N-terminal sequence obtained for purified AtPAP26 with its deduced full-length amino acid sequence [87,89,103,126]. Furthermore, these bioinformatic predictions do not resolve how potential PTMs influence protein localization and/or function. Secreted AtPAP12, AtPAP25, and AtPAP26 of Pi-deprived *Arabidopsis* all have signal peptides that are cleaved at an identical site, starting after a conserved arginine residue [87,89,103]. Therefore, it is imperative that that online programs predicting protein properties such as subcellular localization be followed up using “top-down” experimental approaches to give direct evidence of the presence and effect of PTMs.

## 5. The Effect of Biotic and Abiotic Stresses on the Extracellular Proteome

Plants, as sessile organisms, are constantly subjected to both abiotic and biotic stressors, such as changes in their environment and attack by herbivores and pathogenic microbes. To overcome these stresses, plants have evolved a multitude of physical and biochemical strategies. The structural support and physical barrier provided by the cell wall can act as the first line of defense against biotic and abiotic stresses, with changes in cell wall structure often being facilitated by PTMs. Understanding the mechanism underlying the maintenance of cell wall integrity has captured the interest of researchers as it has implications in developing stress-resistant crop plants; as such, several reviews regarding cell wall integrity have been published in recent years [127,128,129,130,131]. To investigate stress-induced proteome modifications, 2D-PAGE or gel fee approaches coupled with MS have become powerful tools, as exemplified in Figure 3 [68,132]. Responses of the cell wall proteome to both biotic and abiotic stresses will be briefly considered here.

### 5.1. Biotic Stresses

Biotic stress involves stress invoked by other living organisms, with pathogenic bacteria or fungi being the most common biotic stresses imposed upon plants or plant cells when investigating CWPs [26,69]. The goal of these bacteria and fungi is to break through the cell wall to infiltrate the cell and/or apoplast; as such, a common response to such a biotic stress may include strengthening the cell wall and producing elicitors to deter the pathogen or fungi, as well as to warn the plant under attack, along with plants growing in the area, of the threat.

In order to overcome the physical barrier that is the cell wall, the majority of pathogens produce cell wall degrading enzymes [133].Various components within the cell wall, such as CWPs and polysaccharides, exhibit altered functions in response to these elicitors of pathogen attack. When *Arabidopsis* suspension cell cultures were treated with chitosan, a linear polysaccharide that mimics a fungal pathogen attack, there was evidence of defense and stress responses in the extracellular proteome [6]. Several tyrosine phosphorylation and dephosphorylation events occurred among CWPs, suggesting that signal transduction and/or modification of enzyme activity was taking place. Similarly, when *Arabidopsis* suspension cells were exposed to fungal elicitors, the abundance of four CWPs, being an endochitinase (*At3g12500*), a polygalacturonase inhibiting protein (*At5g06870*), a putative receptor-like protein kinase (*At1g78850*), and a probable apospory-associated protein (*At4g25900*), was significantly increased [26]. Endochitinase randomly hydrolyses internal β-1,4-linkages of GlcNAc polymers of chitin, a common component of fungal cell walls. Polygalacturonase inhibiting proteins inhibit the activity of fungal endopolygalacturonases, which degrade the polysaccharide-rich cell wall of the infected plant; these secreted inhibitor proteins can differentiate between fungal or plant endopolygalacturonases [26,134]. Cell wall associated receptor-like kinases (WAKs), a novel protein kinase subfamily, play a crucial role in signal transduction and plant defense. WAKs are considered to be one of the most critical components for cell wall-cytoplasm communication during biotic stress [135,136]. As such, the proteins identified as being up-regulated during times of stress appear to be directly involved in relieving said stress. However, the role of many proteins, such as the probable apospory-associated protein remains unclear [26,137].

A well-known early response to biotic stress is the rapid generation of reactive oxygen species, also known as a respiratory or oxidative burst. Several cell wall-localized peroxidases have been identified as being involved in catalyzing the oxidation of reactive oxygen species during an oxidative burst [138]. The production of reactive oxygen species can aid in mediating the response to biotic stress by strengthening the cell wall via the cross-linking of structural proteins [7,11]. When treated with fungal elicitors, two structural cell wall proteins from *Glycine max* underwent oxidative insolubilization, becoming cross-linked [29]. The cross-linking of cell wall components makes the cell wall tougher, preventing further intrusion by pathogens or fungi.

Other stress-inducible CWPs can also act to modify components of the cell wall, such as polysaccharides and pectins. Polysaccharides are modified throughout the life of the cell wall, during cell growth, development, cell-to-cell interaction, as well as when interacting with the environment; common modifications include methylation, acetylation, and feruloylation. Decreasing the level of polysaccharide acetylation in *Arabidopsis* and *Brachypodium* significantly increased plant resistance to the necrotrophic fungi *B. cinerea* and *B. sorokiniana* [139]. Conversely, increasing the methyl esterification of pectins aids in the response to cell wall degrading enzymes, as it escalates the production of methanol and oligogalacturonides, which are elicitors involved in the early stages of the pathogen attack response [133]. Moreover, pectin de-esterification improves the function and structure of the mechanism employed by pathogens and nematodes to survive in the host cell [133].

### 5.2. Abiotic Stresses

Abiotic stresses encompass environmental conditions that challenge the plant’s ability to grow optimally, including temperature fluxes, strong or UV light, heavy metals, salinity, drought, nutrient deficiency, and hypoxia [140]. Understanding cell wall metabolism in response to these stresses is complex, and can vary based upon factors such as species, genotype, age, and the type and duration of stress that the plant is exposed to; these factors make it difficult to find common patterns among different plant species [141]. The cell wall contains a structurally and enzymatically diverse group of proteins which are believed to play an important role in the cell wall extensibility and plasticity during abiotic stress. These proteins include xyloglucan hydrolases, endo-1,4-β-d-glucanase, expansins, pectin methylesterase, polygalacturonase, pectin/pectate lyase-like, pectin acetylesterase, and cell wall-bound peroxidases [141].

Conduction of signals in response to environmental change begins in the cell wall, with pectin-bound WAKs transducing the signal from the cell wall to the plasma membrane to the cytoplasm [142,143]. Xyloglucan hydrolases, in response to the signalling cascade, can modify xyloglucans in two separate ways. Xyloglucan endo-transglucosylase (XET) activity results in the non-hydrolytic splitting of xyloglucan chains, with the subsequent addition of the short chain of xyloglucan into another xyloglucan chain, ultimately loosening the cell wall [144]. Transcript abundance of XET was found to increase in response to heat stress during fruit ripening on a grapevine (*Vitis vinifera*) [145]. An increase in XET abundance and/or activity could result in greater flexibility of the cell wall, therefore permitting adaptation to heat stress [141]. Interestingly, an isozyme of XET is also induced in rice during cold stress, and overexpressing XET in transgenic *Arabidopsis* can increase the plant’s tolerance to cold stress [141]. Likewise, the activity of cell wall-localized peroxidases can contribute to changes in the flexibility of the cell wall, as they are thought to aid in loosening and lignification, as well as in scavenging hydrogen peroxide during stress [146,147,148,149]. Peroxidases can contribute to loosening the cell wall via the generation of free radicals, which act to cleave polysaccharides [7]. Conversely, they can help to strengthen the cell wall by the oxidative cross-linking or coupling of aromatic molecules, using hydrogen peroxide as the electron acceptor, to form polymers such as lignin and suberin [7,11]. Cell wall-bound peroxidases show marked induction during times of abiotic stress, evidencing the importance of peroxidase activity under these conditions [149,150,151,152].

Whether biotic or abiotic, the imposed stress can induce dramatic alterations within the cell wall proteome that aid in plants acclimation to unavoidable stressful conditions. Signal transduction cascades beginning within the apoplast can trigger changes in cell wall flexibility to mediate the stress at hand by the action of a diverse number of enzymes. Gaining a full understanding of the molecular pathways involved in these alterations, including associated CWP PTMs, will likely contribute toward future biotechnological efforts of engineering stress-resistant crops and trees. Protein PTMs are essential for seemingly all aspects of normal cell wall development, including cell shape and morphology, CWP localization, protein-protein interactions, signal transduction, and CWP activity. While the basic mechanisms underlying these PTMs are fairly well understood, particularly in non-plant eukaryotic systems such as yeast and mammalian cells, there remains a need to reveal the specific timing and function of these modifications within plant cell walls. This will require comprehensive analyses of potential target proteins identified by global CWP studies to fully tease apart the specific details and functions of PTMs of individual CWPs under varying growth stages and environmental conditions.

## 6. Concluding Remarks

Although CWPs only comprise 5%–10% of the total cell wall mass, they are critical in determining cell size and shape, cell wall integrity, and cellular responses to environmental cues. Defining the CWPs that compose the extracellular proteome has proven to be a difficult task, although new techniques and technologies continue to evolve, allowing more CWPs to be identified and characterized. Large-scale, “bottom-up” proteomic studies have provided many targets for more directed, downstream analyses that will illuminate a detailed picture of how CWPs function and interact within the extracellular environment. Future research needs to include targeted, “top-down” proteomic analyses, whereby CWPs fully purified as active, native enzymes can be identified and obtained for the detailed elucidation of their biological and biochemical functions, thereby facilitating their functional categorization. Of particular interest is the identification of CWP PTMs, which can exert great influence over protein localization, activity, and interactions with other cell wall components and proteins. Knowing how and when CWPs are post-translationally modified will provide crucial insights into how plant cells respond to and transduce extracellular abiotic and biotic stress stimuli. With a more complete and integrative knowledge of how CWPs are regulated, it may be possible to engineer crop plants that are more resistant to the many unavoidable stresses that they are frequently exposed to in their ever-changing environment.

## Figures and Tables

**Table 1:** Methods of extracting the *Arabidopsis thaliana* extracellular proteome. Non-destructive methods include those that preserve the integrity of the plasma membrane, whereas destructive methods involve the homogenization of tissues.

	Type of Proteome	Proteome Source	Method Employed	Reference
**Non-Destructive:**	Secretome	Culture medium of liquid-cultured seedlings	Collection of culture filtrate	[64]
	Secretome	Culture medium of suspension cell cultures	Collection of culture filtrate	[65]
	Secretome	Culture medium of hydroponically-grown plants	Collection of culture filtrate	[66]
	Secretome	Culture medium of suspension cells treated with salicylic acid	Collection of culture filtrate	[67]
	Subset of the secretome	Culture medium of suspension cells grown with or without phosphate	Collection of culture filtrate	[68]
	Subset of the secretome	Culture medium of suspension cells infected with *Pseudomonas syringae*	Collection of culture filtrate	[69]
	Cell wall proteome	Suspension cell culture	Sequential washing of intact cells with salt solutions	[68,70]
	Cell wall proteome	Suspension cell culture	Wash intact cells with salt solution	[69]
	Apoplastic fluid proteome	Leaves	Vacuum infiltration	[71,72]
	Subset of the apoplastic fluid proteome	Seedlings treated with oligogalacturonides	Vacuum infiltration	[73]
**Destructive:**	Cell wall proteome	Suspension cell culture	Salt extraction of purified cell walls	[8]
	Cell wall proteome	Suspension cell culture	Salt extraction of purified cell walls	[6]
	Cell wall proteome	Etiolated hypocotyls	Salt extraction of purified cell walls	[74]
	Cell wall proteome	Etiolated hypocotyls	Salt extraction of purified cell walls	[9]
	Subset of cell wall proteome	Suspension cells treated with fungal elicitors	Salt extraction of purified cell walls	[26]
	Cell wall glycoproteome	Etiolated hypocotyls	Lectin affinity chromatography	[75]
	*N*-glycoproteome	Mature stems	Lectin affinity chromatography	[76]
	Glycosylphosphatidyl- inositol anchored proteome	Callus cells	Phospholipase C treatment of purified membrane fraction	[77]
	Arabinogalactan proteins proteome	Liquid-cultured etiolated seedlings	Yariv precipitation	[78]
